# Re-analysis of whole blastocysts after trophectoderm biopsy indicated chromosome aneuploidy

**DOI:** 10.1186/s40246-019-0253-z

**Published:** 2020-01-13

**Authors:** Zhanhui Ou, Zhiheng Chen, Minna Yin, Yu Deng, Yunhao Liang, Wenjun Wang, Yuanqing Yao, Ling Sun

**Affiliations:** 1 Center of Reproductive Medicine, Guangzhou Women and Children’s Medical Center, Guangzhou Medical University, Guangzhou, 510623 People’s Republic of China; 2 Reproductive Medicine Centre, Department of Obstetrics and Gynaecology, Sun Yat-Sen Memorial Hospital, Sun Yat-Sen University, No.107 Yanjiangxi Road, Yuexiu Qu, Guangzhou, 510120 Guangdong People’s Republic of China; 3 Department of Obstetrics and Gynecology, Chinese PLA General Hospital, Beijing, 100853 People’s Republic of China

**Keywords:** Preimplantation genetic testing for chromosomal structural rearrangement, Next-generation sequencing, Mosaic, Discordance, Primary chromosomal rearrangement

## Abstract

**Background:**

To compare the concordance between trophectoderm (TE) analysis and whole blastocyst analysis of embryos from chromosomal structural rearrangement (SR) carriers.

**Method:**

Sixty-three abnormal blastocysts identified by preimplantation genetic testing for chromosomal structural rearrangement (PGT-SR) were included. The whole blastocysts were processed through multiple displacement amplification cycle and sequenced for 24-chromosome aneuploidy screening by next-generation sequencing (NGS). The sequencing results were compared with those of TE biopsy from the same blastocysts and the primary chromosomal rearrangement of the couples.

**Results:**

Analysis of the 63 blastocysts showed 68% (43/63) complete concordance between TE sequencing analysis and whole blastocyst results. Approximately one third (20/63, 32%) of the sequencing results showed some level of discordance between the two samples. Of these, 14% (9/63) of the embryos were identified as euploid after whole blastocyst sequencing. Among them, seven blastocysts were classified as chromosome mosaicism (five whole chromosomes, two segmental) after TE analysis, while two displayed non-SR related segmental changes in the TE biopsy. Of the original analyses, 70% (44/63) of findings were associated with the primary parental chromosomal rearrangement, while 30% (19/63) had no association.

**Conclusions:**

TE biopsy with NGS for PGT-SR is an efficient strategy to identify embryos suitable for transfer. While there was a high concordance between TE and whole blastocyst chromosome results, some embryos classified as mosaic in the original analysis and therefore unsuitable for transfer were reclassified as chromosomally balanced. To maximize the number of embryos available for PGT-SR patients, we suggest that embryos with mosaic non-SR chromosomal rearrangement should be stored and considered for transfer after appropriate counseling.

## Background

Preimplantation genetic diagnosis (PGD) for embryo chromosome assessment was first employed nearly three decades ago in 1990 for embryo analysis of two couples at risk of transmitting a sex linked disease [1]. The technology proved successful in identifying suitable embryos and quickly gained acceptance, with subsequent wide use in both chromosome and gene disorder diagnosis. SR carriers, who are at high risk of generating chromosomally unbalanced embryos (and pregnancies), have a valuable tool to assist in the identification of balanced embryos for selected transfer. This resulted in a marked reduction in miscarriages and improved liveborn rates for these patients.

Recently, in order to promote uniformity in test applications in the assisted reproduction field, preimplantation genetic testing (PGT) was re-classified into PGT-A for aneuploid assessments, PGT-M for monogenetic disease, and PGT-SR for SR. In the PGT procedure, biopsy sample and the subsequent analysis methods were considered two key points to ensure the accuracy of PGT results.

Compared to the original one-cell biopsy from day 3 embryos , TE biopsy (5–10 cells per time) has been shown to provide more accurate diagnosis and a lower allele drop-out rate [2, 3]; and the biopsy itself appears to have little or no negative impact on subsequent embryo implantation [4]. TE biopsy combined with NGS for PGT-SR has now become the most widely used approach in clinics throughout the world [5–7]. Furthermore, the NGS approach offers the option of quantitative chromosome analysis and the ability to report mosaic embryos.

Embryo mosaicism is defined as the presence of more than one distinct cell line within an embryo [8]. The blastocyst stage embryo essentially consists of two different cell lineages: TE and inner cell mass (ICM), with a derivation point believed to be possibly as early as day 1 or 2 of growth. This means that any errors in chromosome segregation during mitosis can result in chromosomal differences between these two lineages or potentially, even differences within a single cell line.

A recent study by Huang et al. re-analyzed three separate TE pieces and ICM as a single piece in 51 aneuploid blastocysts. The results showed eight (16%, 8/51) blastocysts with some level of discordance among the ICM and the three TE pieces [9]. Victor et al. employed a similar approach and found the concordance rate was 97% (93/96) in case of whole chromosome aneuploidy, but the rate decreased to 43% (3/7) in case of segmental aneuploidy [5]. A further study re-analyzed previously tested abnormal blastocysts and results showed the concordance was 50% (3/6) [6]. Tortoriello et al. [6] went so far as to express great concern about the discordances they observed when different platforms were used for embryo analysis and concluded that it was important to better understand the techniques and possibly restricting use to only some categories of patients.

In this study, we wanted to explore the concordance between the primary TE biopsy with the remaining blastocyst as a whole. Sixty-three blastocysts which were considered abnormal after first round PGT-SR were re-tested by the same NGS method but this time as a whole embryo. The clinical significance of our findings is discussed.

## Methods

### Ethics

This study was approved by the Reproductive Medical Ethics Committee of Guangzhou Women and Children’s Hospital. Written informed consent was obtained from each couple.

### Embryo resources

A total of 63 abnormal blastocysts were donated by 18 couples enrolled in our clinic for IVF/PGT-SR. The average maternal age in this study was 31.3 years. The indications for PGT-SR were carriers of balanced translocations, Robertsonian translocations, or a chromosome inversion. Among the 63 blastocysts, there were seven blastocysts (from seven couples) that were considered unsuitable for transfer because of an elevated mosaic state. All of these seven couples had normal blastocysts to transfer and had an ongoing clinical pregnancy or a healthy baby prior to initiating this follow-up analysis.

### Embryo culture and biopsy

Intracytoplasmic sperm injection (ICSI) was performed after oocyte retrieval, and embryos were cultivated in sequential G1/G2 media (Vitro Life, Sweden). Biopsy was performed on day 5 or day 6 according to the blastocyst grade on that day [10].

All of the blastocysts were subjected to trophectoderm-cell-biopsy by laser and 5–10 TE cells were biopsied. After biopsy, blastocysts were cryopreserved using vitrification according to the manufacturer’s protocol (ARSCI Inc., Canada) and then stored in liquid nitrogen.

### NGS protocol for the TE biopsy

The multiple displacement amplification (MDA, Qiagen) DNA amplification system was used for whole genome amplification (WGA) to generate sufficient DNA for analysis. MDA reactions were incubated at 30 °C for 8 h and then heat-inactivated at 65 °C for 3 min according to the manufacturer’s (Qiagen, Germany) protocol.

The Illumina MiSeq platform was used for NGS, and approximately 1.5 million fragments of amplified DNA from each TE biopsy were sequenced. An on-instrument computer performed primary and secondary data analysis to align the reads to a reference genome. PGXcloud cloud server (available at http://www.pgxcloud.com/) was used to analyze the chromosomal copy number variants (CNVs) (Jabrehoo, China). All profile reports were analyzed independently by two laboratory technicians. In the event of any differences in final assessment between the technicians, a consensus was reached after further team discussion.

### Criterion for mosaic embryo

Using NGS, embryos with less than 20% aneuploidy in the TE sample were classified as euploid; those between 20 and 80% were reported as mosaic, while those over 80% were classified as aneuploid; this is in conformation with the current PGDIS guidelines and others [7].

### Re-analysis of abnormal whole blastocysts

**Fig. 1:**
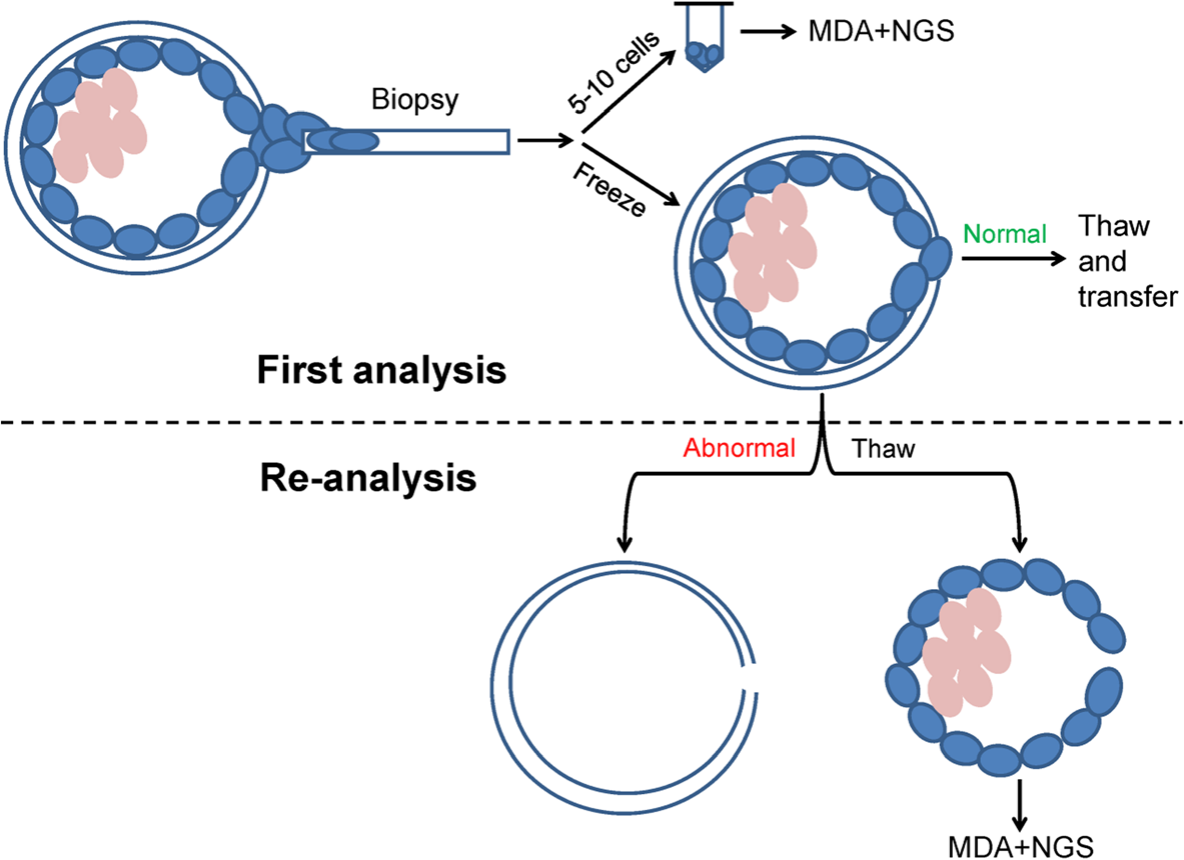
The flow diagram of this study. The pink cycles represent the inner cell mass; the blue cycles represent the trophectoderm cells. First analysis: 5–10 TE cells were biopsied for the multiple displacement amplification (MDA) and next-generation sequencing (NGS). Re-analysis: pellucid zone were removed. The whole blastocysts cells were collected and performed MDA and NGS

After PGT-SR, 63 “abnormal” blastocysts were thawed and incubated. When the thawed blastocyst had expanded, the zona pellucida was removed. Cells from the remaining whole blastocyst were collected as a single specimen, and DNA was amplified by MDA and then analyzed by NGS as for the original TE biopsy pieces (Fig. 1).

## Results

The detailed NGS results of TE and whole blastocysts are presented in Tables 1, 2, and 3 (concordant results between TE and whole blastocyst) and Tables 4, 5, and 6 (discordant results between TE and whole blastocyst).

**Table 1:** Concordant results, related to parental chromosomal rearrangement (*N* = 32)

Embryo number	Gardner grade	Primary chromosomal rearrangement	First analysis of trophectoderm cell	Re-analysis of whole blastocysts
1	IVCC	46,XY,t(9;15)(q22;q22)	del(9)(q22.1), dup(15)(q22.2)	del(9)(q22.1), dup(15)(q22.2)
2	VBB	46,XY,t(9;15)(q22;q22)	dup(9)(q22.33), del(15)(q22.2)	dup(9)(q22.33), del(15)(q22.2)
4	IIIBC	46,XY,t(9;15)(q22;q22)	dup(9)(q22.1), del(15)(q22.2)	dup(9)(q22.1),del(15)(q22.2)
5	VBA	46,XY,t(9;15)(q22;q22)	del(9)(q22.2),dup(15)(q22.2)	del(9)(q22.2),dup(15)(q22.2)
6	VBC	46,XY,t(9;15)(q22;q22)	dup(9)(q22.1), del(15)(q22.2)	dup(9)(q22.1), del(15)(q22.2)
7	VCB	45,XY,der(13;14)(q10;q10)	dup(14)	dup(14)
8	IIIBB	46,XY,t(1;2)(q25;q14.2)	del(1)(q25.3),dup(2)(q14.3)	del(1)(q25.3),dup(2)(q14.3)
9	IVAB	46,XY,t(1;2)(q25;q14.2)	dup(1)(q25.3), del(2)(q14.3)	dup(1)(q25.3), del(2)(q14.3)
12	IIIBC	46,XY,t(1;2)(q25;q14.2)	dup(1)(q25.3), del(2)(q14.3)	dup(1)(q25.3), del(2)(q14.3)
13	VBC	46,XX,t(11;18)(p13;p11.2)	del(11)(p14.3), dup(18)(p11.31)	del(11)(p14.3), dup(18)(p11.31)
14	VBC	46,XX,t(11;18)(p13;p11.2)	del(11)(p14.3), dup(18)(p11.31)	del(11)(p14.3), dup(18)(p11.31)
16	IIIBB	46,XX,t(1;8)(q25;p21)	dup(1)(q24.3), del(8)(p23.1)	dup(1)(q24.3), del(8)(p23.1)
20	IIIBC	46,XX,inv(4)(p12q21.1)	dup(4)(q12), del(4)(q26)	dup(4)(q12), del(4)(q26)
23	IVAB	46,XY,t(18;22)(q23;q11.2)	dup(22)	dup(22)
24	IVAB	46,XY,t(18;22)(q23;q11.2)	dup(18), del(22)(q11.1-q11.21)	dup(18), del(22)(q11.1-q11.21)
25	IIIAA	46,XY,t(18;22)(q23;q11.2)	dup(18), dup22(q11), del(22)(q11.1-q11.21)	dup(18), dup22(q11)(31 Mb), del(22)(q11.1-q11.21)
26	IVCB	46,XY,t(18;22)(q23;q11.2)	del(22)(q11.1-q11.21)	del(22)(q11.1-q11.21)
29	IVBA	46,XY,t(18;22)(q23;q11.2)	dup(18), del(22)	dup(18), del(22)
30	VBB	46,XY,t(18;22)(q23;q11.2)	dup(18), del(22)(q11.1-q11.21), dup(22)(q11.21)	dup(18), del(22)(q11.1-q11.21), dup(22)(q11.21)
31	IVAB	46,XX,t(3;7)(q27;q22)	dup(3)	dup(3)
35	IVAB	46,XX,t(7;8)(p13;p23)	del(7)(p13), dup(8)(p23.2)	del(7)(p13), dup(8)(p23.2)
36	IIIAA	46,XX,t(7;8)(p13;p23)	del(7)(p13), dup(8)(p23.2)	del(7)(p13), dup(8)(p23.2)
37	IIIAB	46,XX,t(7;8)(p13;p23)	dup(7)(p13), del(8)(p23)	dup(7)(p13), del(8)(p23)
42	VCB	46,XY,t(1;15)(q25.1;q25)	del(1)(q24.2), dup(15)(q21.2)	del(1)(q24.2), dup(15)(q21.2)
47	IVBC	46,XY,t(1;15)(q25.1;q25)	del(1)(q24.3), dup(15)(q25.1)	del(1)(q24.3), dup(15)(q25.1)
48	VBB	45,XX,psu dic(15;22)(q12;q11.2)	dup(15)	dup(15)
51	VCB	45,XX,psu dic(15;22)(q12;q11.2)	del(22)	del(22)
52	VBC	45,XX,psu dic(15;22)(q12;q11.2)	dup(15)	dup(15)
55	IVBC	46,XX,t(5;18)(q33;q21)	del(5)(q34), dup(18)(q21.32)	del(5)(q34), dup(18)(q21.32)
56	IVBB	46,XY,t(3;7)(p25;p14)	del(3)(p24.3), dup(7)(p14.2)	del(3)(p24.3), dup(7)(p14.2)
57	IVBC	46,XY,t(3;7)(p25;p14)	dup(3)(p24.2), del(7)(p14.2)	dup(3)(p24.2), del(7)(p14.2)
58	VBB	46,XY,t(3;7)(p25;p14)	dup(3)(p24.3), del(7)(p14.2)	dup(3)(p24.3), del(7)(p14.2)

**Table 2:** Concordant results, partly related to parental chromosomal rearrangement (*N* = 5)

Embryo number	Gardner grade	Primary chromosomal rearrangement	First analysis of trophectoderm cell	Re-analysis of whole blastocysts
18	IIIAB	46,XX,inv(4)(p12;q21.1)	del(4)(p15.32), dup(4)(q26), *del(16)*	del(4)(p15.32), dup(4)(q26), *del(16)*
22	IVCB	46,XY,t(18;22)(q23;11.2)	*mos del (2)(q21.2)(50%)*, dup(22)	*mos del (2)(q21.2)(30%)*, dup(22)
28	VBC	46,XY,t(18;22)(q23;11.2)	*del(17)(q24.3)*, dup(22)	*del(17)(q24.3)*, dup(22)
33	IVAC	46,XX,t(3;7)(q27;q22)	dup(3)(q24), del(7)(q21.13), *del(14)*	dup(3)(q24), del(7)(q21.13), *mos del (14)( 50%)*
43	VBC	46,XY,t(1;15)(q25.1;q25)	del(15) , *dup(16)*	del(15) , *dup(16)*

Italicized data indicate the abnormality not associated with the primary chromosomal rearrangement

**Table 3:** Concordant results, not related to parental chromosomal rearrangement (*N* = 6)

Embryo number	Gardner grade	Primary chromosomal rearrangement	First analysis of trophectoderm cell	Re-analysis of whole blastocysts
17	VBB	46,XX,t(1;8)(q25;p21)	dup(4)	dup(4)
44	VBC	46,XY,t(1;15)(q25.1;q25)	del(3)(q22.2)	mos del(3)(q22.2)(40%)
45	IVCB	46,XY,t(1;15)(q25.1;q25)	del(16)	del(16)
60	IVBB	46,XY,inv(22)(q11.2q13.3)	del(14), dup(22)	del(14), dup(22)
62	IVBC	45,XY,der(13;14)(q10;q10)	del(21)	del(21)
63	IVCB	46,XX,t(11;19)(q13.1;q13.1)	dup(22)	dup(22)

**Table 4:** Discordant results, abnormality in the trophectoderm was related with the parental chromosomal rearrangement (*N* = 7)

Embryo Number	Gardner grade	Primary chromosomal rearrangement	First analysis of trophectoderm cell	Re-analysis of whole blastocysts
3	IIIBB	46,XY,t(9;15)(q22;q22)	*dup(10)(p13)*, del(15)	del(15)
11	VICB	46,XY,t(1;2)(q25;q14.2)	*dup(2)(q12.3)*	del(1)(q25.3), dup(2)(q14.3)
21	IVBB	46,XY,t(18;22)(q23;11.2)	*dup(13)*, dup(22)(q11.2)	dup(22)(q11.2)
27	IVCB	46,XY,t(18;22)(q23;11.2)	dup(22)(q11), *mos del(X)(p11.22)(70%)*	dup(22)(q11)
34	IVBC	46,XX,t(3;7)(q27;q22)	del(2)(p25.2), *dup(2)(p25.2-p24.1)*, *dup(3)(q26.2),* del(7)(q21.13)	del(2)(p25.2), dup(3)(q26.2), del(7)(q21.13r)
38	IIIBC	46,XX,t(7;8)(p13;p23)	*mos dup(4)(40%)*, mos dup (7)(p13)(40%) , del(8)(p23), *mos dup (13)(40%), mos dup(16)(40%), mos dup(17)(40%)*	dup(7)(p13), del(8)(p23)
54	VBB	46,XY,t(3;7)(p13;q21.2)	mos dup (7)(40%)	46,XN

Discordant results are in italics

**Table 5:** Discordant results, abnormality in the trophectoderm was unrelated with the parental chromosomal rearrangement (*N* = 7)

Embryo number	Gardner grade	Primary chromosomal rearrangement	First analysis of trophectoderm cell	Re-analysis of whole blastocysts
19	IVBB	46,XX,inv(4)(p12q21.1)	*mos del(3)(60%)*	del(3)(q28)
32	IVBA	46,XX,t(3;7)(q27;q22)	*dup(16)(p13.11)*	46,XN
40	IIIAA	46,XY,inv(20)(p12q13.1)	*mos dup(7)(50%) , mos dup(10)(60%),* del(22)	del(22)
41	IVBC	46,XY,inv(20)(p12q13.1)	*mos del (7)(q31.1)(50%), dup tetra(7)(q36.1)*	mos del (7)(q21q22)(50%), del(7)(q36.1)
49	VBB	45,XX,psu dic(15;22)(q12;q11.2)	*dup(20)(q11.23)*	46,XN
50	IVBB	45,XX,psu dic(15;22)(q12;q11.2)	*del*(*4)*	del(4)(q28.1)
53	VBC	45,XX,psu dic(15;22)(q12;q11.2)	*mos dup(2)(p21)(40%), mos dup(6)(70%)*	dup(6)

Discordant results are in italics

**Table 6:** Discordant results, the only abnormality being mosaicism unrelated to the parental SR (*N* = 6)

Embryo number	Gardner grade	Primary chromosomal rearrangement	First analysis of trophectoderm cell	Re-analysis of whole blastocysts
10	VBB	46,XY,t(1;2)(q25;q14.2)	mos dup(7)(40%)	46,XN
15	IVAA	46,XX,t(1;8)(q25;p21)	mos del(14)(40%), mos dup(22)(50%)	46,XN
39	IIIBC	46,XX,t(7;8)(p13;p23)	mos del(9)(50%)	46,XN
46	IVCB	46,XY,t(1;15)(q25.1;q25)	mos dup(16)(30%), mos dup(17)(40%), mos dup(21)(30%), mos del(X)(40%)	46,XN
59	VBB	46,XY,inv(22)(q11.2q13.3)	mos del(8)(q11.1)(50%)	46,XN
61	VCB	45,XY,der(13;14)(q10;q10)	mos del(6)(p21.1)(40%)	46,XN

### Concordance between TE and whole blastocyst results

Comparing the sequencing results between the TE and the whole blastocysts, we found 68% (43/63) were concordant (Tables 1, 2, and 3), and 32% (20/63) displayed some level of discordance (Tables 4, 5, and 6). Fourteen percent (9/63) of the blastocysts identified as abnormal after TE sequencing were subsequently deemed euploid after the remaining whole blastocyst was tested (embryos in Tables 4, 5, and 6). Among them, seven blastocysts were classified as chromosome mosaicism (five whole chromosomes aneuploidy, two segmental chromosome aneuploidy) after TE analysis (embryos in Tables 4 and 6), while two displayed non-SR related segmental changes in the TE biopsy (embryos in Table 5).

### Relationship of the aneuploidy to the primary chromosomal disorder

We found that 70% of abnormal blastocysts (44/63) had an abnormal segment copy number related in a direct way to the original parental chromosomal rearrangement (Tables 1, 2, and 4). Of these 44 blastocysts, 32 of had only the translocation/inversion-related changes in the TE (Table 1).

Of the remaining embryos, 19 (Tables 3, 5, and 6) TE results showed aneuploidies that were unrelated to the original parental SR, while only 6 (Table 3) of these blastocysts showed concordant TE/blastocyst changes. The 8 (embryos in Tables 5 and 6) embryos that were discordant were assessed as euploid when the blastocyst was analyzed. In 6 (Table 6) of these now euploid embryos, the original TE result was middle level (30–50%) mosaic, either whole chromosome or segmental regions. The other two (embryos 32 and 49 in Table 5) embryos had segmental changes in the TE unrelated to the SR being tested for and not evident in the blastocyst.

## Discussion

TE biopsy combined with NGS for PGT-SR is now widely used in clinics around the world for identifying embryos suitable for transfer, so concordance rate between TE with ICM is important. Previous studies have re-analyzed abnormal blastocysts to evaluate the concordance between TE and ICM and reported variable correlations [6, 7, 9]. Chromosomally normal ICM is important, because it is this which will develop into the fetus and a chromosomally balanced liveborn. TE is also important since it is from these cells that placental trophoblast lineages arise, making it key for successful embryo implantation and consequent successful pregnancy outcome [11, 12].

Our results showed 68% of results were totally concordant with that of TE, whereas 32% of TE biopsies had some element of discordance with the whole blastocyst results. Several reasons may be responsible for this discordance, with chromosomal mosaicism being the most widely accepted. Mosaicism due to mitotic non disjunctions is reportedly affecting 30–40% of blastocyst-stage embryos [13–17]. Given the limits of detection for embryo mosaicism (> 10–20%), the number of embryos reported as mosaic in some groups seems to be at odds to experimental results, while it disappears in many cases on re-analysis of the same embryo. With whole abnormal blastocyst testing, any low-level mosaicism would be diluted by the higher proportion of normal cells and so give a clearer idea on what the embryo is as a whole. A number of researchers questioned the validity of using percentage aneuploidy in describing the embryo in terms of its ploidy status and hence its suitability for transfer. It would be fanciful to believe that the person performing the biopsy always takes the mosaic region leaving behind the euploid embryo, and so it leads to the question of whether there may also be another explanation for apparent mosaic findings.

A second reason potentially responsible for the observed discordance may due to the technical aspects of the WGA itself and the analysis method. Compared to TE biopsy, the whole blastocyst provides more templates for WGA, which could lead to less bias and unlikely results in mosaic large unexpected segmental deletion or duplication profiles, found in TE samples. Li et al. compared different WGA methods (SurePlex, MALBAC and MDA) on the genome coverage and bias; they found a number of additional copy number variations (CNVs) were identified after MDA, these CNVs not being present in the original genomic DNA samples [18]. This phenomena was suspected to be due to biases in the hexamer random priming which MDA-WGA employs [19]. Likewise, it can be suggested that choice of WGA may impact on the biases that may be present after high level amplification, affecting regions differently according to starting DNA levels.

The NGS technology may count for the third reason. NGS has the ability to detect these small segmental chromosome imbalances and mosaicism sensitively [20] and so may be a victim of its own technical sensitivity. This study used the same WGA and NGS analysis for both TE and blastocyst and so some potential variables were removed. The unbalanced translocation products from the patient with the 46, XY, t (18;22) (q23;11.2) consistently failed to identify the small region (~ 4 Mb) duplicated or lost on chromosome 18. This may be a mapping issue or possibly an algorithm affect, but it does highlight the importance of understanding the limitations of any analysis method in terms of expected results and their interpretations. It also raises the issue of apparent lack of understanding of meiotic non-disjunction and outcomes in some of the literature reports on mosaic embryos [14, 16], where problematic segmental unbalanced pairs were often not identified.

Classifying the profiles by their relationship with the original parental chromosomal rearrangement, we found 70% of the abnormal blastocysts tested had imbalances that were associated with non-disjunction of the primary translocation chromosomes, while the others had unrelated malsegregations. In PGT-SR, an appreciable quantity of the blastocysts is expected to be abnormal segregates, based on current understandings of crucifix formation during meiosis and subsequent non-disjunction chromosome separation. In the cohort of embryos with changes associated to the primary chromosomal disorders (*N* = 44), only one (2%) embryo was mosaic in TE analysis, but unbalanced in the whole blastocyst result. This is an unusual result and possibly reflects the impact of some of the algorithms used in smoothing and normalizing chromosome copy number, since it was in the more heavily amplified TE biopsy that the mosaic half state was observed. This high concordance level leads us to conclude that the current practice of discarding embryos unbalanced for the SR regions was appropriate, with little likelihood of discarding otherwise good quality embryos.

Of high clinical interest were the six mosaic non-SR chromosomal rearrangement TE results (four whole chromosomes, two segmental chromosomes), subsequently diagnosed as euploid in the blastocysts. This finding may have importance in clinical practice. Healthy babies are born after transferring mosaic embryos [21–23], and case studies suggest that the degree of mosaicism identified in the original TE biopsy was a poor predictor of ongoing pregnancy and miscarriage compared with euploid embryos [24], leaving little to make a transfer decision on.

Our finding in mosaicism may have importance in clinical practice, since the transfer of mosaic blastocysts is still controversial on clinic risk management and/or difficult in counseling. It could be said that three classes of embryos now exist after preimplantation in genetic screening: euploid, aneuploid, and mosaic aneuploid [25]. Web-based questionnaires about the extent of mosaic aneuploidy embryos reveals nearly two thirds of clinical practices believe that mosaic aneuploid embryos should be stored for potential therapeutic use after extensive and appropriate patient counseling [8]. Other authors have suggested that patients should be encouraged to undergo another cycle to obtain euploid embryos, rather than transferring a mosaic embryo [26]. In our study, all of the six embryos with mosaic non-SR chromosomal rearrangement were verified as euploid in whole blastocyst analysis. Therefore, we now propose that such embryos could be stored as a backup for implantation. This can be even more important in PGT-SR cases where often a large majority of embryos are found to be unbalanced for the parental segments leaving few if any for transfer consideration. These could be transferred after appropriate counseling.

There are some limitations in this study. Firstly, only abnormal embryos were used, which eliminated the possibility of investigating the false negative rate. Secondly, there were relatively few embryos available for this analysis and so more mosaic blastocysts could be analyzed in this manner to better understand the nature of mosaic results and implications for their retention and transfer.

In conclusion, TE biopsy combined with NGS for PGT-SR was seen to be an efficient strategy in our clinic to select embryos suitable for transfer with essentially complete concordance between TE and the whole blastocyst for the SR chromosomes. As has been observed by other groups, some embryos originally diagnosed as mosaic for whole chromosome aneuploidy, or segmental chromosome imbalance, were actually euploid. We can suggest therefore that, especially for PGT-SR cases (and possibly in cases where for various reasons limited embryos are otherwise available), mosaic embryos with segmental imbalances or mosaic whole chromosomes that are unrelated to the primary chromosomal rearrangement could be stored as a backup for euploid embryos and considered for transfer after appropriate counseling.

## Data Availability

The datasets used and/or analyzed during the current study are available from the corresponding author on reasonable request.
